# Neuromodulation with transcranial direct current stimulation contributes to motor function recovery via microglia in spinal cord injury

**DOI:** 10.1038/s41598-024-69127-7

**Published:** 2024-08-04

**Authors:** Ryotaro Oishi, Ikuko Takeda, Yukihito Ode, Yuya Okada, Daisuke Kato, Hiroaki Nakashima, Shiro Imagama, Hiroaki Wake

**Affiliations:** 1 Department of Orthopedic Surgery, Nagoya University Graduate School of Medicine, 65 Tsurumai-Cho, Showa-Ku, Nagoya, 466-8550 Japan; 2 Department of Anatomy and Molecular Cell Biology, Nagoya University Graduate School of Medicine, 65 Tsurumai-Cho, Showa-Ku, Nagoya, 466-8550 Japan; 3 Division of Multicellular Circuit Dynamics, National Institute for Physiological Sciences, Myodaiji, Okazaki 444-8585 Japan; 4 Center for Optical Scattering Image Science, Kobe University, Kobe, Japan; 5 Department of Physiological Sciences, Graduate University for Advanced Studies, SOKENDAI, Shonan, Hayama, Kanagawa 240-0193 Japan; 6 Core Research for Evolutional Science and Technology, Japan Science and Technology Agency, 4-1-8 Honcho, Kawaguchi, Saitama 332-0012 Japan

**Keywords:** Glial biology, Neuroimmunology

## Abstract

Spinal cord injury (SCI) is damage or trauma to the spinal cord, which often results in loss of function, sensation, or mobility below the injury site. Transcranial direct current stimulation (tDCS) is a non-invasive and affordable brain stimulation technique used to modulate neuronal circuits, which changes the morphology and activity of microglia in the cerebral cortex. However, whether similar morphological changes can be observed in the spinal cord remains unclear. Therefore, we evaluated neuronal population activity in layer 5 (L5) of M1 following SCI and investigated whether changes in the activities of L5 neurons affect microglia-axon interactions using C57BL/6J mice. We discovered that L5 of the primary motor cortex (corticospinal neurons) exhibited reduced synchronized activity after SCI that correlates with microglial morphology, which was recovered using tDCS. This indicates that tDCS promotes changes in the morphological properties and recovery of microglia after SCI. Combining immunotherapy with tDCS may be effective in treating SCI.

## Introduction

Spinal cord injury (SCI) is a debilitating condition characterized by impaired spinal cord function due to primary trauma, such as traction or spinal cord compression^1^*.* The consequences of SCI extend beyond motor impairments to include severe pathologies such as urinary dysfunction, gastrointestinal disorders, and reproductive impairments^2^. SCI incidence in Japan increased from 40.2 cases per million individuals in the early 1990s to 49 per million individuals in 2018^3,4^. Previous studies have reported the extensive contribution of axonal degeneration and its recovery in SCI; however, growing evidence suggests that glial cells, particularly microglia, are crucial in the recovery of axonal degeneration and the extent of damage^5–7^.

Microglia, the primary immune cells in the central nervous system, participate in the phagocytosis of dead tissue, release of pro-inflammatory cytokines and chemokines, and formation of a boundary with astrocytes in glial scars, negatively affecting axonal regeneration^8–12^. Additionally, dysfunctional neurons in the primary motor cortex (M1) following SCI cause the reorganization of neuronal circuits^13^. Microglia have been shown to modulate their behavior in response to neuronal activity, thereby influencing synaptic and neuronal circuit activities^14^. Therefore, abnormal dynamics in microglia may be influenced by an impaired brain neuronal network, which affects motor function recovery. Microglia accumulation on hyperactive neurons reduces epilepsy activity and exhibits neuroprotective functions^15^. Moreover, they release brain-derived neurotrophic factors that attenuate inhibitory function, contributing to chronic pain in the spinal cord^16^. Furthermore, microglia can strip the synapses during axonal injury^17^, suggesting their role in neuronal circuit activity in SCI. However, the specific changes in microglial behavioral dynamics in the SCI-affected brain region and their implications for motor function recovery remain unclear.

Non-invasive transcranial brain stimulation techniques improve motor function following SCI^18–21^. Therefore, understanding the relationship between neuronal activity changes and microglial dynamics is essential to develop novel therapeutic approaches. Transcranial direct current stimulation (tDCS) is a non-invasive transcranial brain stimulation technique that uses low-intensity continuous electrical currents to activate neurons and regulate cerebral cortex excitability^22,23^. Direct stimulation-induced activation of the cortical neurons is used to treat depression, chronic pain, and neurological disorders such as Parkinson’s disease^24^. The effect of tDCS on neuronal activity lasts for 1–3 h^25,26^ and stimulates voltage-gated Ca^2+^ channels. Although tDCS itself does not alter NMDA receptor activity during short stimulation, induction of long-lasting effects requires NMDA receptors and which modulate neuronal plasticity^27^. It has been shown that anodal tDCS enhances and cathodal stimulation reduces excitability, thus regulating motor function. Additionally, it is an affordable therapeutic technology used to modulate spinal cord neuronal circuits^28–30^. tDCS on the motor cortex induces changes in the spinal network at the lumbar enlargement^28–30^. Following SCI, tDCS can promote motor function in patients with complete paralysis^31^. Moreover, its combination with rehabilitation is a promising approach to enhancing functional recovery^32^. Furthermore, tDCS regulates neuronal cell activity and induces calcium surges in astrocytes, leading to changes in the morphology and activity of astrocyte-microglial interactions in the cerebral cortex^33^, thereby altering cortical plasticity^34^. However, whether similar morphological changes can be observed in the spinal cord remains unclear.

Here, we aimed to determine the mechanism of therapeutic action of tDCS after SCI, focusing on the involvement of neuronal circuit organization and microglia in the spinal cord. This study contributes to developing therapeutic strategies for SCI by revealing the relationship between neuronal activity and microglial dynamics.

## Results

### Neuronal populational activity in layer 5 of M1 after SCI

**Figure 1:**
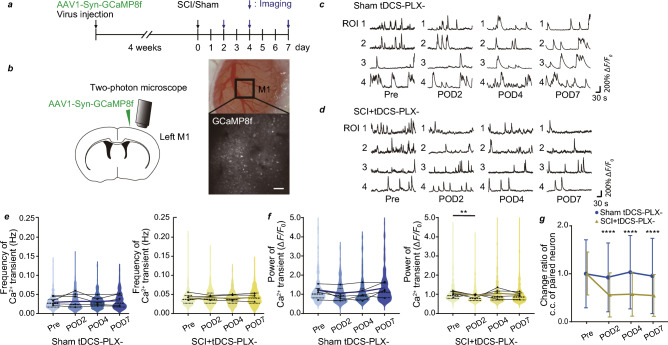
Neuronal activity changes in layer 5 (L5) of the primary motor cortex after spinal cord injury (SCI). (**a**) Experimental scheme. (**b**) Green calcium (Ca^2+^) sensitive fluorescent protein (GCaMP8f)-coded adeno-associated virus (AAV) was injected in L5 of the primary motor cortex (M1). Scale bar: 100 µm. (**c**) Typical traces of Ca^2+^ transients of pre-operation (Pre), post-operation day 2 (POD2), POD4, and POD7 in the Sham tDCS − PLX− group and (**d**) SCI + tDCS − PLX− group. (**e**) Frequency of Ca^2+^ transients of Pre, POD2, POD4, and POD7 in the Sham tDCS − PLX− and SCI + tDCS − PLX− groups. Each dot represents the average data for an individual mouse. (**f**) Power of Ca^2+^ transients (also see “Methods”) of Pre, POD2, POD4, and POD7 in the Sham tDCS − PLX− and SCI + tDCS − PLX− groups. Each black circle represents the average data for an individual mouse. g. Standardized correlational changes in paired neurons of Pre, POD2, POD4, and POD7 in the Sham tDCS − PLX− and SCI + tDCS + PLX− groups. n = 5 mice per group (**e**–**g**). Data are shown as mean ± standard deviation (**g**). **p < 0.01 and ****p < 0.0001; Sham tDCS − PLX− and SCI + tDCS − PLX− groups were compared for each day. Statistical analysis was performed using one-way (**e**, **f**) or two-way (**g**) analysis of variance followed by Bonferroni post-hoc tests.

To evaluate the activity of pyramidal tract neurons after SCI, we first visualized neuronal population activity in layer 5 (L5) of M1 which is responsible for generating pyramidal tract neurons following SCI. Adeno-associated virus (AAV)-coded GCaMP8f was injected into the M1 4 weeks before the SCI or sham surgery (Fig. 1a). In vivo Ca^2+^ imaging of the M1 region using two-photon microscopy was performed to evaluate spontaneous activity 2 days (POD2), 4 days (POD4), and 7 days (POD7) after SCI and compared that with the sham-operated group (Fig. 1b–d). We measured the frequency and the power of Ca^2+^ transients. Neither the frequency nor the power of Ca^2+^ transients showed any detectable changes between timepoints after SCI both in the sham-operated (Sham tDCS − PLX−) and SCI (SCI + tDCS − PLX−) groups (Fig. 1e,f). However, the synchrony of Ca^2+^ transients, measured using cosine correlation, was significantly reduced on POD2, compared with pre-operation levels in the SCI + tDCS − PLX− group. This reduction persisted until POD7 if normalized with pre-operation levels in SCI + tDCS − PLX− group (Fig. 1g).

### Microglia–axon interaction in the spinal cord after SCI

**Figure 2:**
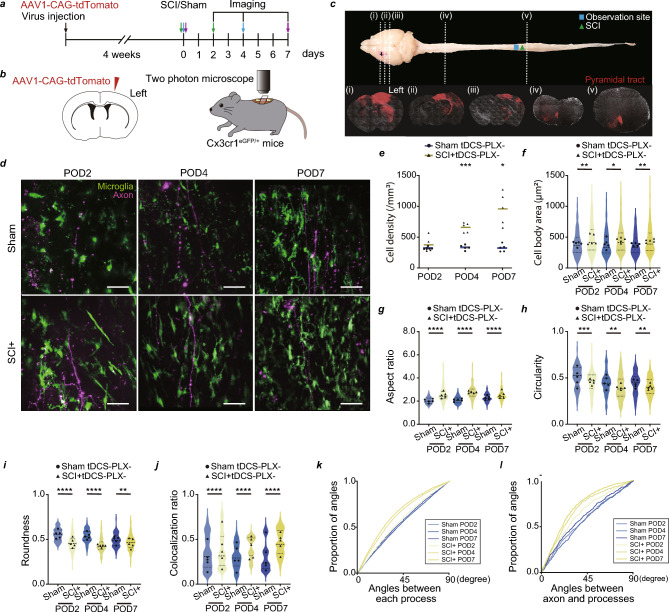
Changes in microglial dynamics and interaction between microglia and the pyramidal tract axons after spinal cord injury (SCI). (**a**) Experimental scheme. Each mouse underwent in vivo imaging once at POD2 (green arrow), POD4 (blue arrow) or POD7 (purple arrow) after SCI or Sham operation. (**b**) tdTomato expression under the CAG promoter-coded adeno-associated virus (AAV) was injected into the primary motor cortex (M1) of Cx3cr1^eGFP/+^ mice to visualize the microglia and pyramidal tract axons in the spinal cord using in vivo two-photon microscopy. (**c**) Whole mounts of the brain, spinal cord, and coronal sections. Pyramidal tract axons from M1 to the spinal cord in (i) primary motor cortex, (ii) internal capsule, (iii) cerebral peduncle, (iv) cervical pyramidal tract axons, and (v) thoracic pyramidal tract axons are labeled with tdTomato. (**d**) Typical images of microglia–pyramidal tract axon interactions in the spinal cord at post-operation day (POD) 2, POD4, and POD7. Scale bar: 50 µm. (**e**) Density of Cx3cr1+ cells in the Sham tDCS − PLX− and SCI + tDCS − PLX− groups at POD2, POD4, and POD7. (**f**) Cell body area of Cx3cr1+ cells in the Sham tDCS − PLX− and SCI + tDCS − PLX− groups at POD2, POD4, and POD7. (**g**) Aspect ratio, (**h**) circularity, and (**i**) roundness of Cx3cr1+ cells in the sham-operated and SCI groups at POD2, POD4, and POD7. (**j**) Colocalization ratio of axonal and microglial fluorescent intensities in the Sham tDCS − PLX− and SCI + tDCS − PLX− groups at POD2, POD4, and POD7. (**k**) Proportion of Cx3cr1+ cell process angles (p = 2.2E − 16, Sham vs. SCI group at POD2; p = 2.99E − 6, at POD4; p = 0.00835, at POD7). (**l**) Proportion of angles between the Cx3cr1+ cell processes and pyramidal tract axons (p = 0.0293, Sham vs. SCI group at POD2; p = 3.41E − 16, at POD4; p = 2.36E − 6, at POD7). n = 5 mice per group (**e**–**l**). Each black circle and black triangle represent the average data for an individual Sham tDCS − PLX− and SCI + tDCS − PLX− mouse, respectively (**e**–**l**). *p < 0.05, **p < 0.01, ***p < 0.001, and ****p < 0.0001; Sham tDCS − PLX− and SCI + tDCS − PLX− groups were compared for each day. Statistical analysis was performed using two-way analysis of variance followed by Bonferroni *post-hoc* tests (**e**–**j**) and Mardia–Watson–Wheeler test (**k**, **l**).

We next investigated whether the changes in pyramidal tract neurons of L5 neuronal activity could affect microglia–axon interactions in the spinal cord. We used Cx3cr1^eGFP/+^ mice to visualize microglia and injected AAV-coded tdTomato into L5 to visualize the pyramidal tract in the spinal cord. Subsequently, we performed a laminectomy of the apex vertebra (T10) lamina (Fig. 2a–c). We first confirmed that tdTomato-positive axons were in the pyramidal tract of the spinal cord using brain and spinal cord sections (Fig. 2c). Microglia–pyramidal tract interactions were visualized using in vivo two-photon microscopy in the upper area of the injured lesion in the spinal cord (Fig. 2d). Time-lapse imaging was performed for 3 h to visualize their interaction at POD2, POD4, and POD7. Consistent with the results of previous studies, the microglia were activated as their cell density and body area increased (Fig. 2e,f). Additionally, their aspect ratio increased, whereas circularity and roundness decreased, suggesting dense interaction with the pyramidal tract axons after SCI (Fig. 2g–i). Consistently, the colocalization of the time stack image (from 3 h; 0.5 frame/min) significantly increased in the SCI group as POD increased compared with the sham-operated group (Fig. 2j). Additionally, to evaluate the polarity of microglial process movement, we calculated the angles between each process or the axons and processes. The SCI group had a higher proportion of smaller angles than the sham-operated group, indicating their association with pyramidal tract axons (Fig. 2k,l). These results indicate that activated microglia are present along the direction in which the axons project.

### tDCS rescues synchrony of pyramidal tract axonal activity

tDCS promotes the synchrony of neuronal circuits^35^ and is a multimodal therapy used to modulate brain and spinal cord excitations in patients with SCI^19^.

**Figure 3:**
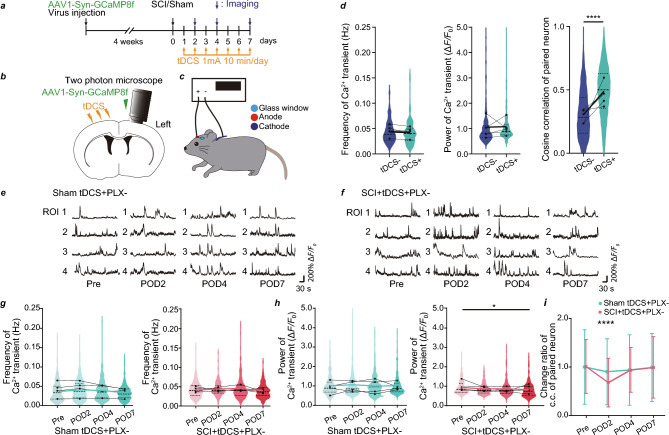
Neuronal activity changes in layer 5 (L5) of the primary motor cortex (M1) after spinal cord injury (SCI) with transcranial direct current stimulation (tDCS). (**a**) Experimental scheme. (**b**) GCaMP8f.-coded adeno-associated virus (AAV) was injected in L5 of M1. (**c**) Experimental scheme of tDCS. The anode was placed over the skull between the eyes, and the cathode was implanted in the neck. (**d**) Frequency of Ca^2+^ transients, power of Ca^2+^ transients, and correlation of paired neuronal activity on post-operation day 7 (POD7) in the tDCS− and tDCS+ sham-operated (Sham) groups (n = 5 mice per group). (**e**) Typical traces of Ca^2+^ transients at pre-operation (Pre), POD2, POD4, and POD7 in the tDCS + sham-operated group and (**f**) tDCS + SCI group. (**g**) Frequency of Ca^2+^ transients at Pre, POD2, POD4, and POD7 in the Sham tDCS + PLX− and SCI + tDCS + PLX− groups. (**h**) Power of Ca^2+^ transients (also see “Methods”) at Pre, POD2, POD4, and POD7 in the Sham tDCS + PLX− and SCI + tDCS + PLX− groups. (**i**) Standardized correlational changes in paired neurons at Pre, POD2, POD4, and POD7 in the Sham tDCS + PLX − and SCI + tDCS + PLX− groups. Each black circle represents the average data for an individual mouse (**d** and **g**–**i**). Data are shown as mean ± standard deviation (**i**). *p < 0.05 and ****p < 0.0001; tDCS− and tDCS+ sham groups were compared (**d**) and Sham tDCS + PLX− and SCI + tDCS + PLX− groups were compared for each day (**g**–**i**). Statistical analysis was performed using unpaired t-test (**d**) and one-way (**g**, **h**) or two-way (**i**) analysis of variance followed by Bonferroni post-hoc tests.

Thus, we hypothesized that tDCS promotes the synchrony of pyramidal tract axonal activity, affecting microglial interaction with the pyramidal tract axons. We demonstrated the effect of tDCS on L5 neurons by injecting AAV-coded GCaMP8f 4 weeks before the sham or SCI operation (Fig. 3a). We applied tDCS at 1 mA for 10 min/day over 7 days to the sham and SCI groups (Fig. 3a–c). tDCS did not increase the frequency and power of Ca^2+^ transients; however, it significantly increased the synchrony of paired neurons (Fig. 3d). Subsequently, we compared the effects of tDCS on the neuronal population activity in the Sham tDCS + PLX− and SCI + tDCS + PLX− groups (Fig. 3e,f). Similar to the results obtained in the Sham tDCS − PLX− and SCI + tDCS − PLX− (Fig. 1e,f), the frequency and power of Ca^2+^ transients did not show any detectable changes in the Sham tDCS + PLX − or SCI + tDCS + PLX− groups (Fig. 3g,h). The SCI + tDCS + PLX− group exhibited a significant reduction in Ca^2+^ transient synchrony only on POD2; however, the difference was much smaller than that observed in the absence of tDCS. Additionally, the synchrony of the Ca^2+^ transients in the SCI + tDCS + PLX− group was significantly rescued on POD4 and POD7 with tDCS (Fig. 3i).

### Microglia interaction with pyramidal tract axons depends on their synchronized activity

**Figure 4:**
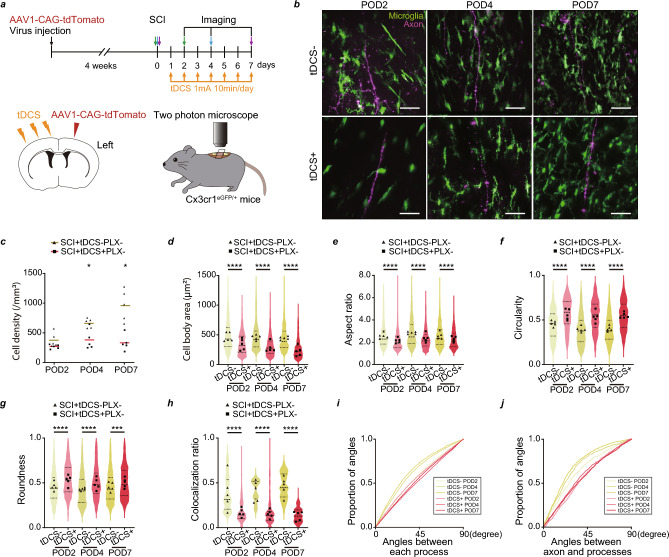
Changes in microglial dynamics and interaction between microglia and the pyramidal tract axons after spinal cord injury (SCI) with or without transcranial direct current stimulation (tDCS). (**a**) Experimental scheme. Each mouse underwent in vivo imaging once at POD2 (green arrow), POD4 (blue arrow), or POD7 (purple arrow) after SCI operation. tdTomato expression under the CAG promoter-coded adeno-associated virus (AAV) was injected into the primary motor cortex (M1) of Cx3cr1^eGFP/+^ mice to visualize the microglia and pyramidal tract axons in the spinal cord using in vivo two-photon microscopy. (**b**) Typical images of microglia–pyramidal tract axon interactions in the spinal cord at post-operation day (POD) 2, POD4, and POD7 after SCI with or without tDCS. Scale bar: 50 µm. (**c**) Density of Cx3cr1+ cells at pre-operation day (Pre), POD2, POD4, and POD7 in the SCI + tDCS − PLX− and SCI + tDCS + PLX− groups. (**d**) Cell body area of Cx3cr1+ cells at Pre, POD2, POD4, and POD7 in the SCI + tDCS − PLX− and SCI + tDCS + PLX− groups. (**e**) Aspect ratio, (**f**) circularity, and (**g**) roundness of Cx3cr1 + cells at Pre, POD2, POD4, and POD7 in the SCI + tDCS − PLX− and SCI + tDCS + PLX− groups. (**h**) Colocalization ratio of axonal and microglial fluorescent intensity at Pre, POD2, POD4, and POD7 in the SCI + tDCS − PLX− and SCI + tDCS + PLX− groups. (**i**) Proportion of Cx3cr1+ cell process angles in the SCI + tDCS − PLX− and SCI + tDCS + PLX− groups (p = 2.20E − 16, SCI + tDCS − PLX− vs. SCI + tDCS + PLX− group at POD2; p = 2.20E − 16, at POD4; p = 1.39E − 5, at POD7). (**j**) Proportion of angles between Cx3cr1+ cell processes and pyramidal tract axons at Pre, POD2, POD4, and POD7 in the SCI + tDCS − PLX− and SCI + tDCS + PLX− groups (p = 3.55E − 4, SCI + tDCS − PLX− vs. SCI + tDCS + PLX− group at POD2; p = 1.71E − 13, at POD4; p = 2.20E − 16, at POD7). n = 5 mice per group (**c**–**j**). Each black circle and black triangle represent the average data for an individual SCI + tDCS − PLX− and SCI + tDCS + PLX− mouse respectively (**c**–**h**). **p < 0.01, ***p < 0.001, and ****p < 0.0001; SCI + tDCS − PLX− and SCI + tDCS + PLX− groups were compared for each day. Statistical analysis was performed using two-way analysis of variance followed by Bonferroni *post-hoc* tests (**c**–**h**) and Mardia–Watson–Wheeler test (**i**, **j**).

We next examined how the synchrony of pyramidal tract axonal activity in the spinal cord affects microglia. We injected AAV-coded tdTomato into the L5 of pyramidal neurons in Cx3cr1^eGFP/+^ mice to visualize the microglia–axon interaction in the spinal cord (Fig. 4a). The mice were subjected to SCI or sham operation. On the day after the operation, 1 mA of tDCS was induced in both groups for 10 min/day over 7 days (Fig. 4a). The SCI mice with tDCS (SCI + tDCS + PLX−) were compared with those without tDCS (SCI + tDCS-PLX−) (Fig. 4b–j) and sham-operated mice with tDCS (Sham tDCS + PLX−) (Fig. S1a–h). SCI induced microglia activation; however, tDCS significantly inhibited SCI-induced microglia activation. The cell density, cell body, and aspect ratio significantly reduced, whereas the circularity and roundness of microglia significantly increased (Fig. 4b–g, Figs. S1a–e, S2a,b). These results suggest minimal interaction between the microglia and pyramidal tract axons. The colocalization ratio of axons with microglial fluorescent intensity was significantly reduced in the SCI + tDCS + PLX− group (Fig. 4h, Figs. S1f, and S2c). Additionally, the proportion of smaller angles was lower in the SCI + tDCS + PLX−  group than in the SCI + tDCS − PLX− group. The proportion of angles was comparable to the Sham tDCS + PLX− group at POD7. It was less associated with the pyramidal tract axons (Fig. 4i,j, Fig. S1g,h). Astrocytes and oligodendrocytes were also analyzed for the effects of tDCS, but no changes were found in their numbers (Fig. S3a–d).

### SCI recovery with tDCS induction depends on the microglia

**Figure 5:**
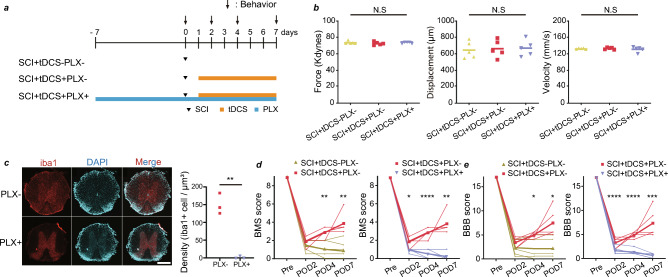
Hindlimb motor function changes after spinal cord injury (SCI) and the effect of transcranial direct current stimulation (tDCS) via microglia. (**a**) Experimental scheme. (**b**) Validation of the Infinite Horizons Impactor device for mouse contusion SCI using actual impact force, calculated displacement measurements, and tip velocity. (**c**) Sample immunohistology sections of the spine with PLX treatment showing microglial loss (stained using Iba1, red). Scale bar: 500 μm. (**d**) Basso Mouse Scale (BMS) score at pre-operation day (Pre), post-operation day (POD)2, POD4, and POD7 in the SCI + tDCS + PLX−/SCI + tDCS − PLX− (left) and SCI + tDCS + PLX−/SCI + tDCS + PLX+ groups (right). Note that both tDCS+ data in these graphs replot the same data. (**e**) Modified Basso, Beattie, and Bresnahan (BBB) score at Pre, POD2, POD4, and POD7 in the SCI + tDCS + PLX−/SCI + tDCS − PLX− (left) and SCI + tDCS + PLX−/SCI + tDCS + PLX+ groups (right). Note that both tDCS+ data in these graphs replot the same data. The thin lines represent the data for individual mice, and the thick lines present the average value. n = 5 mice per group (**b**, **d**, and **e**). *p < 0.05, **p < 0.01, ***p < 0.001, and ****p < 0.0001. Statistical analysis was performed using unpaired t-test (**c**) and one-way (**b**) or two-way repeated (**d**, **e**) ANOVA followed by Bonferroni post-hoc tests.

Consistent with the results of a previous study^36^, tDCS significantly promoted SCI recovery (Fig. 5a–e). We further examined whether the interactions between microglia and axons promoted recovery. We pharmacologically depleted microglia using PLX3397 (PLX, pexidartinib; Fig. 5a–c, and Fig. S4a–e). PLX treatment tended to worsen the Basso Mouse Scale (BMS) and modified Basso, Beattie, and Bresnahan (BBB) scores in the SCI + tDCS + PLX+ and SCI + tDCS − PLX+ groups, suggesting that microglia promote SCI recovery (Fig. 5d,e, and Fig. S4a–e). PLX treatment in the tDCS (SCI + tDCS + PLX+) group significantly impaired BMS and BBB scores, suggesting that microglia contribute to tDCS-dependent SCI recovery (Fig. 5d,e).

## Discussion

In addition to the functional motor impairment caused by the blockade of ascending and descending conduction pathways, brain plasticity occurs in SCI owing to the inhibition of disconnected circuits between peripheral nerves and the brain, which results in functional brain impairments^13^. Here, we discovered that the synchrony of corticospinal neurons in the L5 of the M1 regulates microglial morphology.

Mechanical damage caused by primary injury to the spinal cord, activation of caspase 8 through FAS and TNFR1 activation resulting from secondary injury, activation of caspase 9 through cytochrome C release from the mitochondria, apoptosis and atrophy of neurons caused by cytokines (such as TNFα), and free radical damage have been observed in SCI^37^. These inflammatory responses in the spinal cord after SCI cause reorganization of the corticospinal projection that occurs after SCI. We believe that the primary injury causes desynchronization, and this desynchronization persisted for a week after the injury. Therefore, we consider that both the primary injury and the subsequent inflammation contribute to the persistent desynchronization. This is evidenced by the response of the ventral posterior lateral nucleus to hindlimb stimulation by becoming responsive to forelimb stimulation in a rat thoracic SCI model^38^. Additionally, analyzing high-resolution brain waves revealed differences in the local brain network in SCI^39^. Furthermore, cortical neurons in the primary somatosensory cortex (S1) initially decrease their activity to compensate for the loss of input from damaged pathways, followed by hyperexcitability and paroxysmal discharge^40^, which can lead to neuropathic pain^41,42^. Additionally, increased activity in the M1 area was detected, resulting in the increased plasticity of local neuronal circuits in motor-related areas. This sensitivity can be improved through neuronal regulation via repetitive transcranial magnetic stimulation^43,44^. Therefore, neuronal activity regulation and brain neuronal network reorganization have been considered targets for SCI treatment. Herein, we revealed a decrease in synchronization between the pyramidal tract neurons in M1 during the acute phase after SCI. The effects of such abnormal neuronal activity, especially in changes in synchrony, on the damaged tissue after SCI have not been previously discussed. When synchronization between the pyramidal tract neurons was reduced in the acute phase after SCI, microglia near the injury site exhibited increased cell density, changed into an elliptical shape, and extended processes along the axons. Previous in vitro studies after SCI have reported microglia activation and cell density increase, suggesting that their continuous proliferation forms a dense scar at the boundary between the not-yet-formed astrocytic scar and the fibrous scar by day 7 after injury^12^. The microglia in this study were elliptical in shape and assumed rod-shaped morphology associated with aging, brain infarction, and brain injury. Moreover, they are considered a form of disease-associated microglia^45^. They exhibit changes in orientation along injured neuronal processes^46^, and a similar tendency is possible in SCI. Furthermore, as previously mentioned, microglia regulate the extent of glial scar formation through their activation^47^, which may lead to the loss of their physiological function in monitoring the surrounding environment by extending and retracting their processes^48^.

The activity of the axial directional process observed in this study helps advance the monitoring of damaged axonal tissue. This finding is further supported by the observed increased colocalization rate of microglia with axons. Microglia modulate their dynamics based on neuronal activity^14^*,* and tDCS affects cortical microglial properties such as population and process motility^49^*.* Furthermore, tDCS induces structural synaptic plasticity in the M1 or S1^50^. Additionally, tDCS improved the synchrony between pyramidal tract neurons in the acute phase after SCI.

In a previous study, the combination of neuromodulation and rehabilitation was found to positively interact to maintain neuronal excitability. Furthermore, a combination of neuromodulation and training helps establish the initial activation state and achieve circuit restriction^19^. Even when used alone, tDCS contributes to improvements in the motor functions of the upper and lower extremities after SCI^18,51–54^. tDCS created strong synchronization of neuronal activity in the area surrounding the stimulated region^55^, and this effect might improve the reduced neuronal synchronization after SCI.

In this study, tDCS may have improved abnormal neuronal synchrony and appropriately suppressed abnormal microglial activation, resulting in motor function recovery, similar to the results obtained in a study that administered a colony-stimulating factor 1 receptor antagonist GW2580^56^.

Previous studies showing motor function recovery with GW2580 required drug administration 4 weeks before SCI, rendering it challenging to apply the drug in unexpected SCI cases^57^. Therefore, tDCS, which has immediate effects, can be used as a new treatment method to solve this problem. Furthermore, tDCS combined with microglial manipulation may be a novel therapeutic strategy for SCI.

This study has some limitations. First, although we revealed that tDCS improved neuronal synchrony and inhibited microglial hyperactivity after SCI, the causal relationship and bridging molecular mechanism remain unclear. Second, this study focused only on microglia in the spinal cord and did not evaluate microglia in other regions, including the cerebral cortex. However, microglia in other areas may have contributed to motor function recovery. Despite these limitations, this study demonstrated that neuromodulation using tDCS affects spinal cord microglia and contributes to improved hindlimb function in mice. This finding suggests that tDCS might become a therapeutic modality for SCI via a new mechanism. Further research on optimal stimulation intensity and timing, as well as related molecular mechanisms, may make tDCS an effective treatment for SCI.

## Methods

### Animals

The Animal Care and Use Committee of the Nagoya University Graduate School of Medicine approved the experimental protocols. All methods are reported in accordance with ARRIVE guidelines. A total of 199 mice were used in this study, comprising C57BL/6 (WT) and *Cx3cr1*^eGFP/+^ (C57BL/6 background) mice. The *Cx3cr1*^eGFP/+^ mice expressed enhanced green fluorescent protein (eGFP) under the *Cx3cr1* promoter^58^ and were used to visualize the microglia. All mice used were 6- to 14-week-old males and were provided food and water ad libitum under a 12-h light/dark cycle. We used the mice aged 6–10 weeks for AAV injection, waited for 4 weeks, and then used the mice aged 10–14 weeks for imaging. The area of the cages was 484 cm^2^ and the maximum caging density per cage was five mice. Mice randomly divided into each group: the sham or SCI groups with or without tDCS. Male mice were used because female mice naturally recover locomotor function^59^ and are therefore unsuitable for the experiments. This report was described in accordance with guidelines for reporting of Sex and Gender Equity in Research (SAGER). Mice was sacrificed by isoflurane in accordance with the American Veterinary Medical Association (AVMA) Guidelines for the Euthanasia of Animals. Human resources were not used.

### AAV injection and head plate attachment

The first surgery was performed on mice aged 6–10 weeks. Anesthesia with three types of mixed anesthetic agents was administered intraperitoneally (i.p.): 0.3 mg/kg medetomidine hydrochloride (Domitol, Nippon Zenyaku Kogyo Co., Fukushima, Japan), which is an alpha-2 adrenoceptor agonist; 4.0 mg/kg midazolam (midazolam, Fuji Chemicals Industrial Co., Tokyo, Japan), which is a benzodiazepine derivative; and 5.0 mg/kg butorphanol (Vetorphale, Meiji Animal Health Co., Tokyo, Japan). The skin was disinfected with 70% (w/v) ethanol, and the skull was exposed and cleaned. A 2.5-mm^2^ craniotomy was performed over the left M1, centered at 0 mm anteroposterior and 1.0 mm lateral from the bregma. The neuronal activity in the L5 pyramidal neurons of M1 was visualized by pressure-injecting (IM-300; Narishige, Tokyo, Japan) 2 μL of recombinant AAV encoding the synapsin promoter-driven calcium indicator protein GCaMP8f (9.0 × 10^12^ vector genomes/mL diluted 1:1 in phosphate-buffered saline [PBS]) at three sites in the L5 (at a depth of 550 μm below the cortical surface) using a glass pipette (tip diameter, 30 μm; GDC-1, Narishige). To visualize the pyramidal tract, a 4.0 × 2.0 mm rectangular craniotomy was performed over the left M1 (from 1.0 mm anterior to 3.0 mm posterior and 2.0 mm lateral from the bregma). Subsequently, 2 μL of recombinant AAV encoding the CAG promoter-driven tdTomato protein was pressure injected at three sites (0.4 mm anterior, 1.2 mm lateral, and 0.9 mm posterior; 1.1 mm lateral and 2.0 mm posterior; and 1.5 mm lateral from the bregma) in the L5, using the same technique. Subsequently, the brain surface was covered with a glass window composed of two coverslips (2 and 3.5 mm^2^; Matsunami Glass, Osaka, Japan) and an ultraviolet curable adhesive (NOR-61, Norland Products, Jamesburg, NJ). The edges of the cranial windows were sealed with dental cement (G-CEM ONE; GC, Tokyo, Japan). All AAV solutions were injected for 5 min and incubated for 5 min before withdrawal. After AAV injection, a custom-made headplate was firmly attached to the skull using dental cement. A 2-mm circular silicone pad was attached between the eyes (+ 3.5 mm anterior to bregma) and filled with silicone elastomer (Kwik-Cast, World Precision Instruments, Sarasota, FL) to prevent skull necrosis. This headplate allowed us to securely attach the mouse to a stainless-steel frame to enable craniotomy, subsequent tDCS, and two-photon imaging of neuronal activity in the awake state. After all the procedures, atipamezole (Antisedan; Nippon Zenyaku Kogyo Co.), an alpha 2-adrenergic antagonist that reverses medetomidine effects, was intraperitoneally injected. Subsequently, the mice were individually housed, and the next procedure was initiated approximately 4–5 weeks after the surgical treatments (that is, mice at 10–15 weeks of age).

### SCI

Sham or SCI was induced 4–5 weeks after AAV injection. Under anesthesia with a mixture of three anesthetic agents, the dorsal surface was shaved as much as possible and washed with 70% ethanol to reduce the likelihood of infection. After lidocaine (Xylocaine, Sandoz Co., Tokyo, Japan) was applied to the skin, a dorsal midline incision was made over the T8–T12 level of the mouse spine, and the skin was retracted. The muscles between the spinal and transverse processes were resected using a scalpel to expose the vertebral arch. The spinal processes were then trimmed. The spinal cord was exposed at T10 with laminectomy, and in the SCI mice group, contusion injury was induced on the exposed spinal cord using a force of 70 kdyn with a commercially available SCI device (Infinite Horizon Impactor; Precision Systems and Instrumentation, Lexington, KY), ensuring consistent spinal-cord contusion injury. All procedures were performed on heated surfaces. After completing all procedures, the wound was closed, and atipamezole was intraperitoneally injected. Subsequently, mice were individually housed and urinated manually once daily.

### Spinal fixation surgery

Spinal fixation surgery was performed before spinal imaging. Each mouse underwent only one spinal fixation surgery prior to the relevant imaging. Under anesthesia with three anesthetic agents, the dorsal surface was washed with 70% ethanol. After applying lidocaine to the skin, the dorsal midline was re-cut. Laminectomy was performed on one vertebral arch cranial to a previously operated lamina. Subsequently, the spine was fixed with a fixation device and a glass attachment (cut to fit the exposed spinal cord size) using cyanoacrylate (Aron Alpha; Konishi Co., Osaka, Japan) and dental cement. A handmade water bowl was placed over the glass and attached with cyanoacrylate and dental cement. Atipamezole was intraperitoneally injected after completing all the procedures. Subsequently, two-photon imaging was performed after observing forelimb movement.

### Two-photon imaging

We used a laser scanning system (NIS-Elements; Nikon Instech Co., Tokyo, Japan) and a mode-locked Ti:sapphire Chameleon Ultra II laser (Coherent, Santa Clara, CA) tuned to 950 nm with a water-immersion objective lens (× 16, NA 1.10; Nikon Instech Co.). Fluorescence was separated using a 560-nm dichroic mirror with 500–550 nm (green channel for eGFP fluorescence detection) and a 593-nm mirror with 601–657 nm (red channel for tdTomato fluorescence detection) emission filters. The laser intensity was 3.5–15 mW. Two-photon images of neuronal activity were acquired before and 2, 4, and 7 days after the sham or SCI operations. The imaging sessions were conducted in the L5 of M1 in awake mice at 500–550 μm below the cortical surface. The imaged fields had dimensions of 795.50 × 795.50 μm with a pixel size of 1.55 μm and a resolution of 512 × 512 pixels. Continuous 558-frame imaging was repeated for each imaging field. Each imaging session was conducted 2 h after stimulation for the tDCS group. Microglial and axonal imaging sessions were conducted 50–100 μm below the spinal cord surface 2, 4, and 7 days after the sham or SCI operations. Only one imaging session was performed on each mouse after spinal fixation surgery, and each mouse was sacrificed after imaging. We captured continuous images for 3 h in each file with a 2-min image frame duration (90 frames). The step size for each Z slice was 1 μm, which was driven using a Galvano scanner. The imaged field was 264.90 × 264.90 μm, with a pixel size of 0.517 μm and a resolution of 512 × 512 pixels. Each frame comprised 60–70 Z slices.

### tDCS

tDCS was applied while the mice were awake. A silver wire (Nilaco Co., Tokyo, Japan) with a diameter of 0.5 mm was used as an anode and cathode. The anode silver wire was placed on a sodium chloride-based conductive gel interface (Z101BA; Nihon Koden, Tokyo, Japan) spread over a 2-mm circular area between both eyes, 3.5 mm anterior to the bregma. The cathode was connected to a neck silver wire sutured to the splenius capitis muscle. DC (1.0 mA for 10 min) was applied using an isolated constant current supply.

### Drug administration

For pharmacological microglial ablation, we orally administered the CSF-1R inhibitor PLX3397 (HY-16749, MedChemExpress, Monmouth Junction, NJ; 50 mg/kg) 1 week before the sham or SCI operations until the end of observation^60^.

### Behavioral test

Mice were tested for hindlimb motor function in the open field for 5 min pre-operatively and 2, 4, and 7 days after the sham or SCI operations using the modified BBB and BMS locomotor rating scales^61,62^. The motor function of the hindlimbs was recorded, and two investigators converted it to a score based on the guidelines. The mean of the two scores was used for comparison.

### Tissue collection and immunohistochemistry

Brain and spinal cord slices were used to evaluate the trajectory of tdTomato injected into the L5 of M1 (pyramidal tract), and immunohistochemistry was performed to quantify the effect of PLX3397 on microglial ablation. The mice were anesthetized with ketamine and xylazine and transcardially perfused with PBS (pH 7.4), followed by 4% paraformaldehyde. The fixed brains and spinal cords were equilibrated in a 30% sucrose solution in PBS and cut into 30-μm slices (Leica Microsystems, Wetzlar, Germany). After blocking and permeabilization for 1 h in 5% bovine serum albumin (BSA) and 0.5% Triton X-100 in PBS, the slices were incubated at 4 °C overnight with primary antibodies (rabbit-anti-IBA1, 019-19741, Wako, Osaka, Japan; 1: 1000; chicken-anti-NF1, ab4680, Abcam, Tokyo, Japan, 1:1000; mouse-anti-GFAP, 3670, Cell Signaling Technology, Tokyo, Japan, 1:1000; mouse-anti-APC, ab40778, Abcam, Tokyo, Japan, 1:500) diluted in 5% BSA/PBS. After washing with PBS, the slices were incubated with a secondary antibody (donkey anti-rabbit IgG (H + L) Alexa 594, ab150076, Abcam Japan, Tokyo, Japan,1:500; goat anti-chicken IgG (H + L) Alexa 488, ab150169, Abcam Japan, Tokyo, Japan,1:500; goat anti-mouse IgG (H + L) Alexa 594, ab150116, Abcam Japan, Tokyo, Japan, 1:500) in 5% BSA/PBS at room temperature for 3 h. Imaging was performed using an Olympus FV3000 confocal microscope (Evident, Tokyo, Japan) with a × 10 objective lens (NA 0.30; Olympus, Tokyo, Japan) and a × 60 objective lens (NA 0.90; Olympus, Tokyo, Japan).

### Image analysis

Images were analyzed using ImageJ (version1.53 and 1.54, https://fiji.sc/, National Institutes of Health, Bethesda, MD) and MATLAB (R2019a, MathWorks, Natick, MA). The movies were corrected for focal plane/XY displacement using the ImageJ plugin TurboReg and 3D displacement using descriptor-based series registration.

We estimated the M1 neuronal activity by determining the regions of interest (ROIs) in L5 using an automated algorithm (http://github.com/simonsfoundation/CaImAn) that defined an ROI as a discrete region showing a change in fluorescence at some stage during analysis. Completely silent neurons (and/or those not expressing GcaMP8f) were not detected using this approach. Ca^2+^ transients were detected and analyzed by defining the baseline fluorescence as the 35^th^ percentile of the total fluorescence intensity histogram obtained during the imaging period (*F*_*0*_). Ca^2+^ transient was defined as Δ*F/F*_*0*_ (Δ*F* = *F* − *F*_*0*_), where *F* is the instantaneous fluorescent signal, and Δ*F* exceeded two standard deviations (SDs) of the baseline fluorescence (*F*_*0*_). The frequency of Ca^2+^ transients was calculated as the ratio of the total number of transients during the imaging period (s). The power of each Ca^2+^ transient (Δ*F*/*F*_*0*_) was calculated using the mean squared amplitude of Ca^2+^ during the transient as follows:

$${p}_{x} = \frac{1}{T} \sum_{i=1}^{T}{x}_{i}^{2}$$

where ${x}_{i}$(1 ≤ *I* ≤ T) represents the Ca^2+^ level at each time during the transient of length (T). The correlation coefficient between the activities of two neurons over time was measured in all neurons where Ca^2+^ activities were detected using cosine instead of the commonly used Pearson’s correlation because Pearson’s correlation does not correctly measure the correlation when the time interval without the firing of two neurons is too long. The Pearson’s correlation coefficient (*r*_*p*_) between two Ca^2+^ transients of length *T*, *x*_*i*_ and* y*_*i*_ (1 ≤ *i* ≤ *T*) in two ROIs in the imaging field is given by:

$${r}_{p}= \frac{{\sum }_{i=1}^{T}({x}_{i}- {\mu }_{x})({y}_{i}- {\mu }_{y})}{\sqrt{{\sum }_{i=1}^{T}{({x}_{i}- {\mu }_{x})}^{2}}\sqrt{{\sum }_{i=1}^{T}{({y}_{i}- {\mu }_{y})}^{2}}}$$

where µ_*x*_ and µ_*y*_ represent the average Ca^2+^ fluorescence in the two ROIs. In the equation above, each Ca^2+^ transient average is subtracted from the corresponding Ca^2+^ transient, which is centered. As the Ca^2+^ transients are positive during neuronal firing and approximately zero without neuronal firing, the centered Ca^2+^ transients become negative without neuronal firing. Thus, Pearson’s correlation becomes large if the interval without the firing of two neurons is too long, although neurons do not fire almost simultaneously. The correlation between neurons was measured using the following equation without centering:

$${r}_{c}= \frac{{\sum }_{i=1}^{T}{x}_{i}{y}_{i}}{\sqrt{{\sum }_{i=1}^{T}{x}_{i}^{2}}\sqrt{{\sum }_{i=1}^{T}{y}_{i}^{2}}}$$

${r}_{c}$ can be regarded as the cosine of the angle between two T-dimensional vectors whose elements correspond to *x*_*i*_ and *y*_*i*_. Therefore, it is called the cosine correlation. ${r}_{c}$ ranges from − 1 to 1, and the cosine correlation becomes positively large if two neurons simultaneously fire and becomes close to zero if either neuron does not fire. Additionally, ${r}_{c}$ can be negative because of the negative values of the oscillatory components or noise in the Ca^2+^ transients.

The microglial morphology was assessed by subjecting XYZT images to SD z-projections. The microglial cell body was manually outlined in each frame, and the cell body was extracted using the wand tool, where the cell body was defined as the region with half the intensity from the maximum intensity point; that is, the tolerance of the wand tool was set at half the maximum intensity point. The extracted cell bodies were validated via manual inspection, and those that could not be distinguished from adjacent cells were excluded from the morphological analysis. Then, the number of cell bodies was counted, and the cell density in each field (264.9 × 264.9 µm; 512 × 512 pixels) was calculated for each time. The extracted cell bodies were approximated as ellipses, and their morphological characteristics (area, aspect ratio, roundness, circularity) were assessed using ImageJ. The colocalization ratio between microglia and axons was evaluated by splitting the images into green and red channels and binarizing using the Otsu thresholding method in the ImageJ threshold plugin. Merged areas of the green microglia and red axons were extracted using the image calculator in ImageJ, and the colocalization ratio was calculated as the merged area divided by the axon area.

The tip trajectories of microglial processes were tracked using the Manual Tracking function in ImageJ. The process movements were analyzed for 1 h, and in each process, the vector of the maximum distance coordinates from the starting point was defined as the moving vector of the process within that hour. We validated the polarity of moving processes by calculating the angles (θ) between each process. The angle of a vector (x, y) is expressed as follows:

$$\theta = {\text{tan}}^{-1}\frac{y}{x}$$

The differences between each angle were calculated, and the calculated angles were converted to a range of 0°–90°. The axons were reckoned as a linear approximation connecting each end of the button and were treated as a vector. We assessed the relationship between the axons and microglial processes by calculating the angles between the axons and processes as described above. The cumulative distribution of calculated angles was plotted as the proportion of angles.

### Statistics

Data were analyzed using MATLAB R2019a, R version 4.0.5 (R Foundation, Vienna, Austria), SPSS 28.0.0.0 (IBM, Armonk, NY), and GraphPad Prism 9 (GraphPad Software, San Diego, CA). Statistical significance was set at p < 0.05. All data are presented as mean ± SD. Statistical significance was tested by performing unpaired t-tests, Mardia–Watson–Wheeler test, and analysis of variance (ANOVA) followed by Bonferroni post-hoc tests.

### Ethics approval

The study is reported in accordance with ARRIVE guidelines. All experiments were conducted according to the protocols approved by the Animal Care and Use Committee of the Nagoya University Graduate School of Medicine.

### Study approval

Care of all animals and procedures was approved by Nagoya University.

### Supplementary Information

Supplementary Figures.

## Data Availability

This study did not use any unique reagents. All data reported in this paper will be shared by the lead contact upon reasonable request.

## Code availability

This study does not report on original code. Any additional information required to reanalyze the data reported in this paper is available from the lead contact upon reasonable request.
