# Supplementary material for: Neuromodulation with transcranial direct current stimulation contributes to motor function recovery via microglia in spinal cord injury
Source: Sci Rep. 2024 Aug 4;14:18031. doi: 10.1038/s41598-024-69127-7 (PMC11298548; doi:10.1038/s41598-024-69127-7)
Supplement: Supplementary file 1 — Supplementary Figures. [file 41598_2024_69127_MOESM1_ESM.pdf]

Supplementary Fig. 1

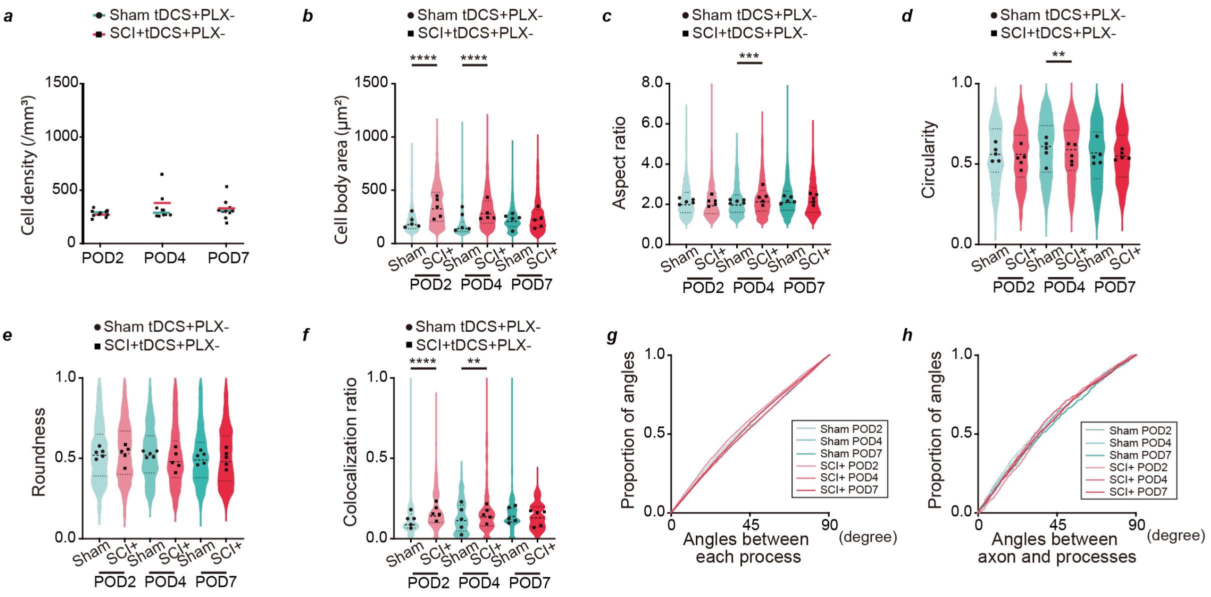

Supplementary Fig 1. Comparison of the properties of microglia and microglial interactions with the pyramidal tract axons between transcranial direct current stimulation (tDCS)+ sham-operated and spinal cord injury (SCI) groups.

a Density of Cx3cr1+ cells in tDCS+ sham-operated (Sham tDCS+PLX−) and SCI groups (SCI+tDCS+PLX−) at post-operation day (POD) 2, POD4, and POD7. The SCI+tDCS+PLX− data in the graph replot the same data in Fig. 4**c**. **b** Cell body area of Cx3cr1+ cells in the Sham tDCS+PLX− and SCI+tDCS+PLX− groups at POD2, POD4, and POD7. The SCI+tDCS+PLX− data in the graph replot the same data in Fig. 4**d**. **c** Aspect ratio, **d** circularity, and **e** roundness (also see Methods) of Cx3cr1+ cells in Sham tDCS+PLX− and SCI+tDCS+PLX− groups at POD2, POD4, and POD7. The SCI+tDCS+PLX− data in the graph replot the same data in Fig. 4**e**, **f**, and **g**. (**f**) Co-localization ratio of the axonal and microglia fluorescent intensities (also see Methods for the binary process) in the Sham tDCS+PLX− and Sham tDCS+PLX− groups at POD2, POD4, and POD7. The SCI+tDCS+PLX− data in the graph replot the same data in Fig. 4**h**. (**g**) The proportion of Cx3cr1+ cells process angles in the Sham tDCS+PLX− and SCI+tDCS+PLX− groups (p = 2.2E−16, SCI+tDCS+PLX− versus

SCI+tDCS+PLX− group at POD2; p = 1.04E−5, at POD4; p = 0.188, at POD7). The SCI+tDCS+PLX− data in the graph replot the same data in Fig. 4**i**. (**h**) Proportion of angles between Cx3cr1+ cell process and pyramidal tract axons in the Sham tDCS+PLX− and SCI+tDCS+PLX− groups (p = 0.487, Sham tDCS+PLX− versus SCI+tDCS+PLX− group at POD2; p = 0.768, at POD4; p = 0.583, at POD7). The SCI+tDCS+PLX− data in the graph replot the same data in Fig. 4**j**. n = 5 mice per each group (**a** to **h**). **p < 0.01, ***p < 0.001, and ****p < 0.0001, Sham tDCS+PLX− and SCI+tDCS+PLX− groups were compared for each day. Statistical analysis was performed using two-way ANOVA followed by Bonferroni *post-hoc* tests (**a** to **f**) and a Mardia–Watson–Wheeler test (**g** and **h**).

Supplementary Fig. 2

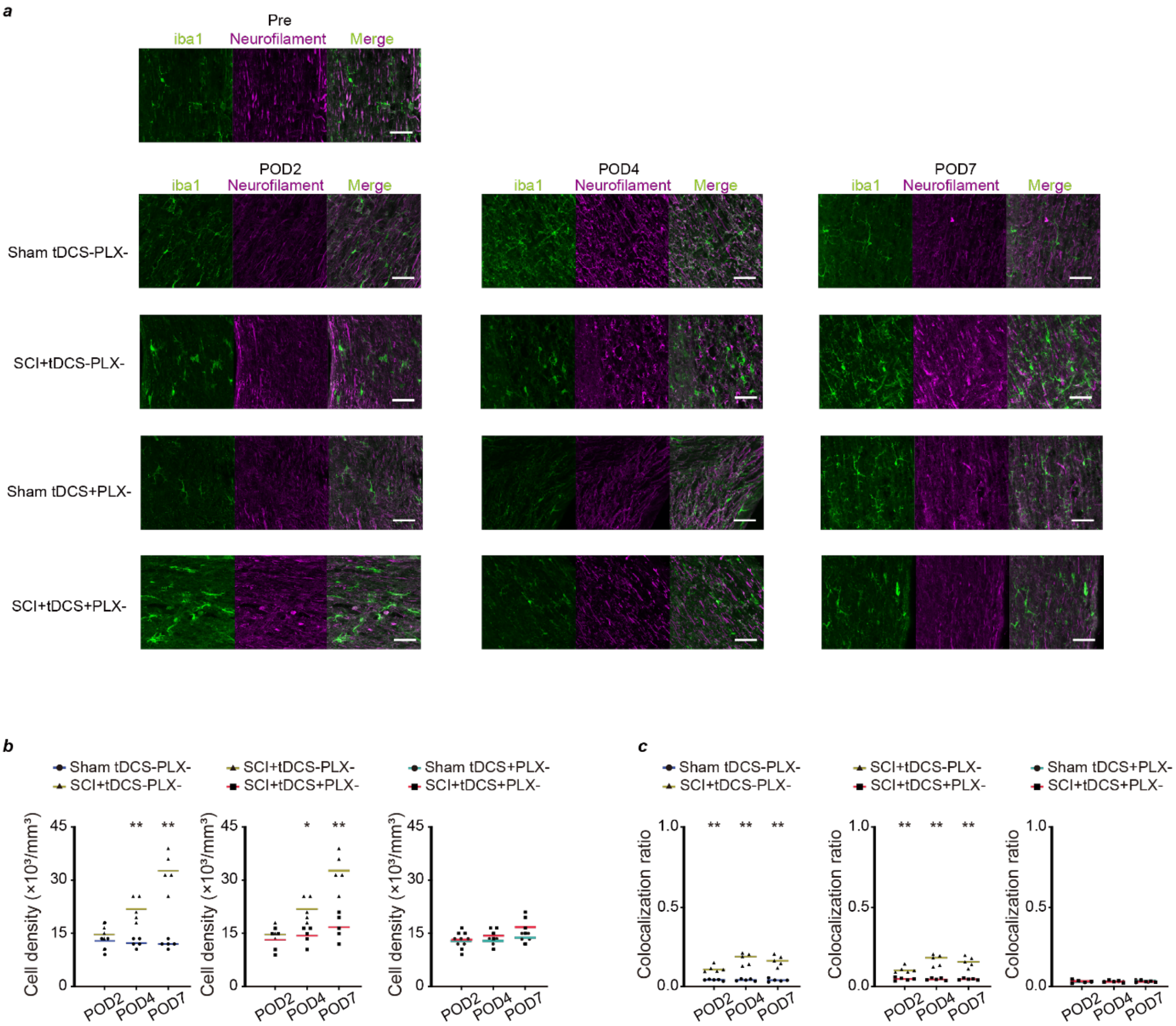

Supplementary Fig 2. Evaluation of changes in microglial dynamics and interaction between microglia and the pyramidal tract axons after spinal cord injury (SCI) by immunohistochemistry.

a Typical images of microglia–pyramidal tract axon interactions in the spinal cord at pre-operation (Pre), post-operation day (POD) 2, POD4, and POD7 after the Sham tDCS–PLX–, SCI+tDCS–PLX–, Sham tDCS+PLX–, and SCI+tDCS+PLX– groups in immunohistochemistry. Scale bar: 50 µm. **b** Density of Iba1+ cells at POD2, POD4, and POD7 in the Sham tDCS–PLX–, SCI+tDCS-PLX-, Sham tDCS+PLX–, and SCI+tDCS+PLX– groups. **c** Colocalization ratio of axonal and microglial fluorescent intensity at POD2, POD4, and POD7 in the the Sham tDCS–PLX–, SCI+tDCS–PLX–, Sham

tDCS+PLX−, and SCI+tDCS+PLX− groups. n = 5 mice per group (**b** and **c**). Each black circle, black triangle, and black square represent the average data for an individual. *$p < 0.05$ and **$p < 0.01$; Sham tDCS−PLX−, SCI+tDCS−PLX−, Sham tDCS+PLX−, and SCI+tDCS+PLX− groups were compared for each day. Statistical analysis was performed using two-way analysis of variance followed by Bonferroni post-hoc tests (**b** and **c**).

Supplementary Fig. 3

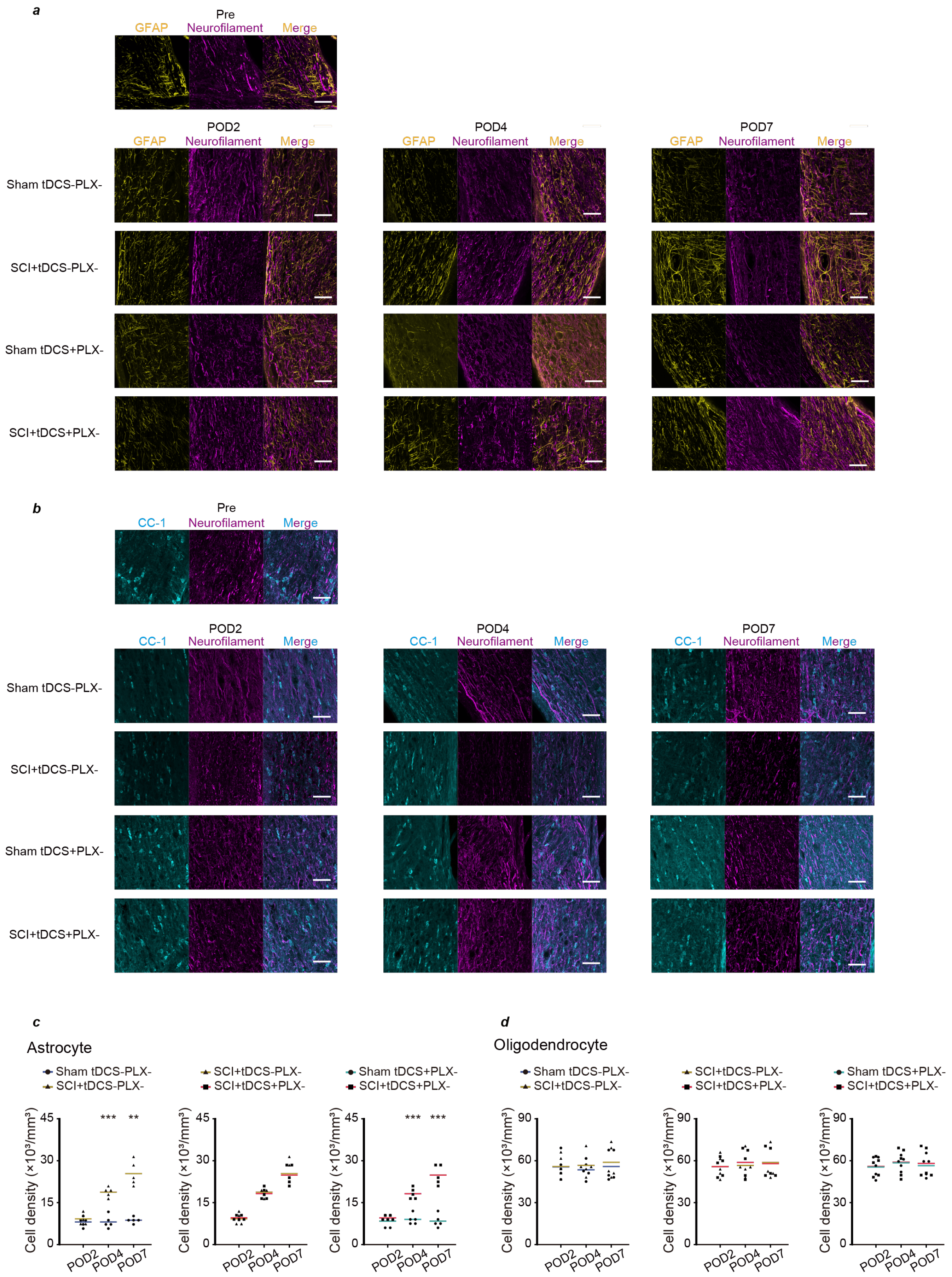

Supplementary Fig 3. Evaluation of changes in astrocyte and oligodendrocyte after spinal cord injury (SCI) by immunohistochemistry.

a Typical images of astrocytes in the spinal cord at pre-operation (Pre), post-operation day (POD) 2, POD4, and POD7 after the Sham tDCS−PLX−, SCI+tDCS−PLX−, Sham tDCS+PLX−, and SCI+tDCS+PLX− groups in immunohistochemistry. Scale bar: 50 μm. **b** Density of GFAP+ cells at POD2, POD4, and POD7 in the Sham tDCS−PLX−, SCI+tDCS-PLX-, Sham tDCS+PLX−, and SCI+tDCS+PLX− groups. **c** Colocalization ratio of axonal and microglial fluorescent intensity at POD2, POD4, and POD7 in the the Sham tDCS−PLX−, SCI+tDCS−PLX−, Sham tDCS+PLX−, and SCI+tDCS+PLX− groups. n = 5 mice per group (b and c). Each black circle, black triangle, and black square represent the average data for an individual. $^{**}p < 0.01$ and $^{***}p < 0.001$; Sham tDCS−PLX−, SCI+tDCS−PLX−, Sham tDCS+PLX−, and SCI+tDCS+PLX− groups were compared for each day. Statistical analysis was performed using two-way analysis of variance followed by Bonferroni post-hoc tests (**b** and **c**).

Supplementary Fig. 4

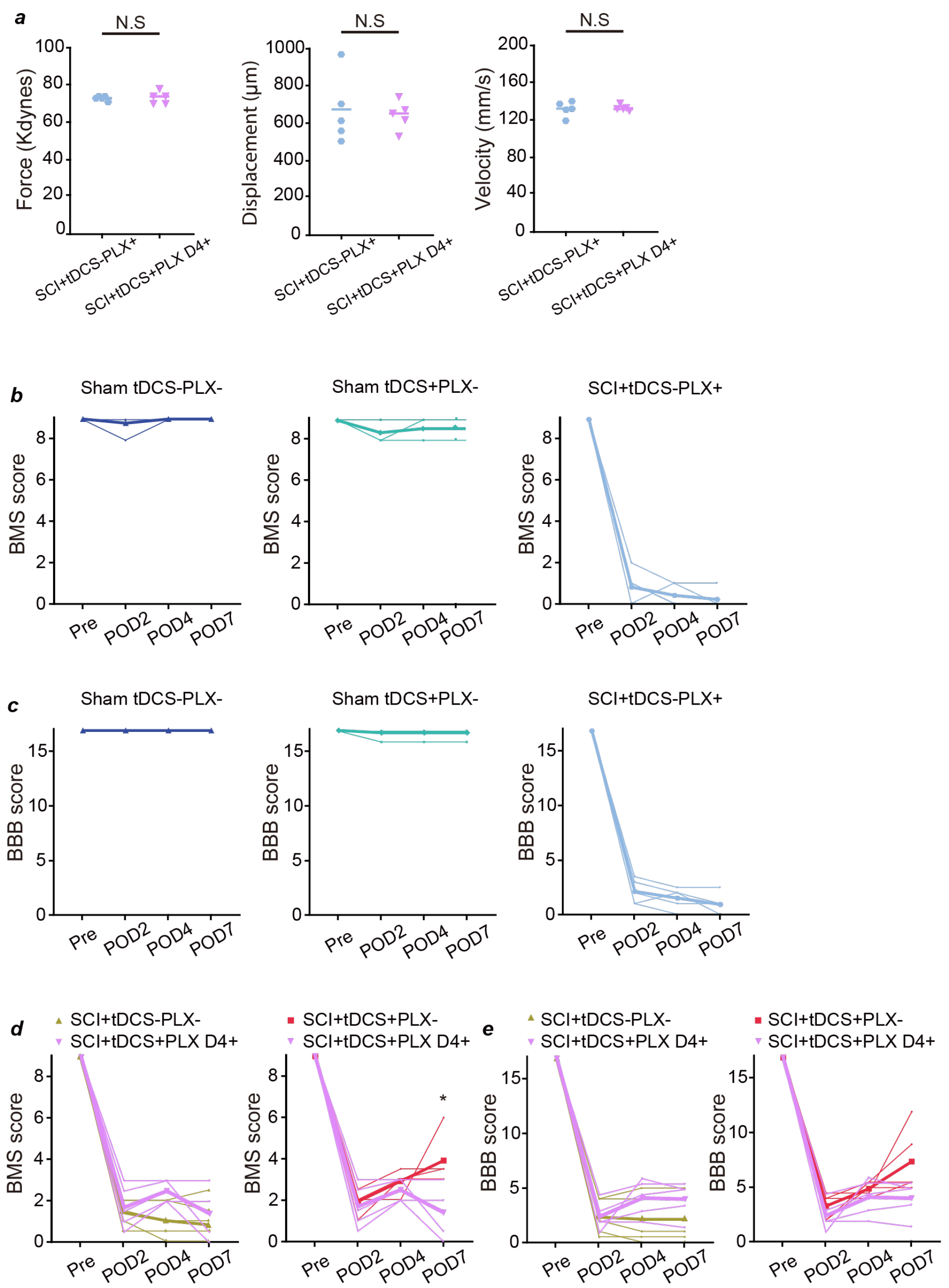

Supplementary Figure 4. Motor function changes under various conditions.

a Validation of the Infinite Horizons Impactor device for mouse contusion SCI using actual impact force, calculated displacement measurements, and tip velocity. **b** Basso Mouse Scale (BMS) score at pre-operation day (Pre), post-operation day (POD)2, POD4, and POD7 in the Sham tDCS–PLX– (left), Sham tDCS+PLX– group (middle), and SCI+tDCS–PLX+ group

after PLX treatment (right). **c** Modified Basso, Beattie, and Bresnahan (BBB) score at Pre, POD2, POD4, and POD7 in Sham tDCS−PLX− group (left), Sham tDCS+PLX− group (middle), and SCI+tDCS−PLX+ group after PLX treatment (right). **d** BMS (left) and BBB (right) score at pre-operation day (Pre), post-operation day (POD)2, POD4, and POD7 in SCI+tDCS−PLX− group, SCI+tDCS+PLX− group, and SCI with tDCS and PLX administered from day 4 (SCI+tDCS+PLX D4+) group. Note that SCI+tDCS−PLX− and SCI+tDCS+PLX− data in these graphs replot the same data in Fig 5**d** and **e**. n = 5 mice per each group (**b** to **d**). The thin lines represent the data for individual mice, and the thick lines present the average value (**b** to **d**).
